# Criteria for the determination of maximal oxygen uptake in patients newly diagnosed with cancer: Baseline data from the randomized controlled trial of physical training and cancer (Phys-Can)

**DOI:** 10.1371/journal.pone.0234507

**Published:** 2020-06-11

**Authors:** Ann Christin Helgesen Bjørke, Truls Raastad, Sveinung Berntsen

**Affiliations:** 1 Department of Sport Science and Physical Education, University of Agder, Kristiansand, Norway; 2 Department of Physical Performance, Norwegian School of Sport Sciences, Oslo, Norway; University of Bourgogne France Comté, FRANCE

## Abstract

**Introduction:**

Maximal oxygen uptake (${\dot{V}O}_{2}\max$) is a measure of cardiorespiratory fitness often used to monitor changes in fitness during and after treatment in cancer patients. There is, however, limited knowledge in how criteria verifying ${\dot{V}O}_{2}\max$ work for patients newly diagnosed with cancer. Therefore, the aim of this study was to describe the prevalence of fulfillment of typical criteria verifying ${\dot{V}O}_{2}\max$ and to investigate the associations between the criteria and the test leader’s evaluation whether a test was performed “to exhaustion”. An additional aim was to establish new cut-points within the associated criteria.

**Methods:**

From the Phys-Can randomized controlled trial, 535 patients (59 ±12 years) newly diagnosed with breast (79%), prostate (17%) or colorectal cancer (4%) performed an incremental ${\dot{V}O}_{2}\max$ test on a treadmill. The test was performed before starting (neo-)adjuvant treatment and an exercise intervention. Fulfillment of different cut-points within typical criteria verifying ${\dot{V}O}_{2}\max$ was described. The dependent key variables included in the initial bivariate analysis were achievement of a ${\dot{V}O}_{2}$ plateau, peak values for maximal heart rate, respiratory exchange ratio (RER), the patients’ rating of perceived exertion on Borg’s scale_6-20_ and peak breathing frequency (*f*_R_). A receiver operating characteristic analysis was performed to establish cut-points for variables associated with the test leader’s evaluation. Last, a cross-validation of the cut-points found in the receiver operating characteristic analysis was performed on a comparable sample of cancer patients (n = 80).

**Results:**

The criteria RERpeak (<0.001), Borg’s RPE (<0.001) and *f*_R_ peak (p = 0.018) were associated with the test leader’s evaluation of whether a test was defined as “to exhaustion”. The cut-points that best predicted the test leader’s evaluation were RER ≥ 1.14, RPE ≥ 18 and *f*_R_ ≥ 40. Maximal heart rate and ${\dot{V}O}_{2}$ plateau was not associated with the test leader’s evaluation.

**Conclusion:**

We recommend a focus on RER (in the range between ≥1.1 and ≥1.15) and RPE (≥17 or ≥18) in addition to the test leader’s evaluation. Additionally, a *f*_R_ peak of ≥40 breaths/min may be a cut-point to help the test leader evaluate the degree of exhaustion. However, more research is needed to verify our findings, and to investigate how these criteria will work within a population that are undergoing or finished with cancer treatment.

## Introduction

A continuously increasing number of people are living with or have survived cancer [1], with most new cases occurring in persons aged 50 years and older [2]. Importantly, although improved treatment strategies have increased survival from cancer [3], most cancer treatments are collectively accompanied with negative effects on healthy cells and tissues [4–6]. Low levels of physical activity in people diagnosed with cancer [7], in combination with side effects from treatments causing injuries to the cardiovascular and muscular system [6, 8–10], are potent reasons for the clinically relevant impairments in cardiorespiratory fitness often observed in cancer treated individuals [11–14].

Patients with cancer are recommended to be as physically active as their abilities and conditions allow before, during and after cancer treatment [15, 16]. However, current exercise recommendations are rather general [17] and do not differ much for patients with cancer compared with the healthy population [18]. Based on a lack of individually tailored physical activity and exercise guidelines (e.g. frequency, intensity, type and time), second-generation trials, where specific exercise prescriptions are being investigated, are needed [19]. To be able to prescribe tailored exercise programs involving endurance training and to evaluate the effect of exercise programs, valid measurements of cardiorespiratory fitness are fundamental. One important challenge with maximal exercise tests in various patient groups, and older adults in general, is whether tests are performed with maximal effort [20]. A consequence of using submaximal test results is prescribing an exercise intensity that is too low. In addition, comparisons within (e.g. comparing different exercise intensities) and between studies is complicated if we rely on biased data [21].

When measuring cardiorespiratory fitness, direct assessment of maximal oxygen uptake (${\dot{V}O}_{2}\max$) is acknowledged as the gold standard [22]. To ensure high validity and reliability of a ${\dot{V}O}_{2}\max$ test (i.e. results can be reproduced), accurate instruments and experienced personnel are important [23]. Different patients and healthy individuals have various levels of experience with exercise and subjective evaluations of their effort. Furthermore, among patients with cancer, the heterogeneity may be even larger because they often are older [2], more unfit [11], and may have comorbidities and side effects like fatigue or pain [4, 24, 25]. Therefore, when assessing such a heterogenetic group of people, objective criteria to support the decision whether a patient with cancer has reached her/his maximal effort (verifying ${\dot{V}O}_{2}\max$) is important [23].

The most widely used objective criteria, a plateau or levelling off in $\dot{V}O_{2}$ with increasing workload, has been extensively debated the last 20–30 years [26–31]. Variations in the number of subjects attaining a ${\dot{V}O}_{2}$ plateau are seen across studies [32], and secondary criteria are also included when verifying ${\dot{V}O}_{2}\max$. The term ${\dot{V}O}_{2}\mathrm{peak}$ (the highest value attained during exercise [33]) is often used when involving exercise-naïve and/or clinical populations, as there is an assumption that these persons seldom reach their highest physiologically attainable value (${\dot{V}O}_{2}\max$) [33]. In the literature, estimated peak heart rate (HR), peak respiratory exchange ratio (RER), post exercise blood lactate (BLa^-^), and self-reported Rating of Perceived Exertion (RPE) on Borg’s scale_6-20_ (or other scales), with a variety of cut-points, are reported as secondary criteria to verify ${\dot{V}O}_{2}\max$ [34, 35]. How close these secondary criteria are associated with ${\dot{V}O}_{2}\max$ is not well validated. Because they all have pros and cons, the criteria and their cut-points have been discussed in the literature [23, 34–36]. Furthermore, there is no consensus on how to apply these criteria in various populations [23], but some suggestions have been made for healthy athletes [34], healthy adult subjects between 20 and 85 years [37], and for overweight or obese adults [38, 39]. It might be challenging to apply these criteria in patients newly diagnosed with cancer, and whether this population have the same physiological responses as other populations is questionable. Nevertheless, the use of well-defined objective criteria in testing newly diagnosed cancer patients is probably more important than in healthy populations because both the patient and test leader might be afraid of pushing towards maximal effort. In addition to the often-used criteria, respiratory frequency (*f*_R_) has been suggested as a valid variable for defining maximal effort [40], but to our knowledge, *f*_R_ has not been used as a criterion in ${\dot{V}O}_{2}\max$ testing. Personal experiences from test-laboratories, in which *f*_R_ has been found to be useful as part of the effort-evaluation of people performing a ${\dot{V}O}_{2}\max$ test, is another rationale for adding this variable as a possible secondary criteria to verify ${\dot{V}O}_{2}\max$.

The test leader’s subjective evaluation whether a ${\dot{V}O}_{2}\max$ test is performed to exhaustion is important when considering the validity of ${\dot{V}O}_{2}\max$ tests. Although evaluations of exertion are based on predefined observations of body language and facial expressions, subjectivity is still part of the test leader’s evaluation. How test personnel give instructions and how they verbally encourage the person being tested are examples of possible biases that may affect the validity of the test results [41]. Submaximal results may occur if the test leader is inexperienced and is too “kind”; meaning that he/she does not motivate the person being tested enough, or even terminates the test before a maximal effort has been reached, of various reasons (e.g. the cancer diagnosis, comorbidities or age). Because of the aforementioned challenges of using the ${\dot{V}O}_{2}$ plateau in the evaluation of whether ${\dot{V}O}_{2}\max$ is reached, we are dependent on experienced and highly skilled test leaders who are able to evaluate whether a test is performed to exhaustion. In the present study we chose this somewhat experimental approach, by giving the test leaders’ evaluation of each ${\dot{V}O}_{2}\max$ test a focus in the statistical analyses.

To our knowledge, there are only one published study where criteria verifying ${\dot{V}O}_{2}\max$ have been investigated within a population of patients diagnosed with cancer [42]. Schneider et al. (2019) investigated how a supramaximal verification bout could be applied in relation to feasibility and whether it could serve as a criterion when verifying ${\dot{V}O}_{2}\max$ in survivors from breast and prostate cancer [42]. The present study will support researchers and test leaders in their decision concerning which secondary criteria to apply when evaluating future ${\dot{V}O}_{2}\max$ tests in newly diagnosed patients with breast, prostate or colorectal cancer. Presumably, not all ${\dot{V}O}_{2}\max$ tests in the future will be performed with an added verification bout. We present the fulfillment of a variety of criteria with different cut-points in our sample of patients. The primary objective was to determine which of the following variables; ${\dot{V}O}_{2}$ plateau, RERpeak, HRpeak, Borg’s RPE and *f*_R_ peak, were associated with the test leader’s subjective evaluation of whether the tests were defined as “to exhaustion”. In addition, cut-points within the associated criteria were established. A second objective was to cross-validate these cut-points in a comparable sample of patients with cancer.

## Methods

### Design and participants

The Phys-Can study was a multicenter randomized exercise trial with a descriptive observational study to be used for comparison [43]. For the intervention trial involving exercise, 600 adults (≥18 years) recently diagnosed with either curable breast, prostate or colorectal cancer scheduled to begin their (neo-)adjuvant therapy in Uppsala, Linköping and Malmö/Lund (Sweden) were included. Exclusion criteria were stage IIIb-IV breast cancer, inability to perform basic activities of daily living, cognitive disorders, severe psychiatric disease or other disabling conditions that might contraindicate high intensity exercise (e.g. severe heart failure, severe chronic obstructive pulmonary disease or orthopaedic conditions), treatment for an additional ongoing malignant disease, BMI<18.5 kg/m^2^ or pregnancy. This main study was performed between March 2015 and November 2018. Full descriptions of the purpose, the design and enrollment of the study are presented elsewhere [43]. The observational study included 102 people following the same eligibility criteria and was performed between September 2014 and February 2015. All persons deemed as eligible by a physician/oncologist were contacted by a member of the research staff who provided verbal and written information about the study. Those who agreed to participate in the study gave their written informed consent before baseline data collection. For the purpose of the present study and analyses performed herein, 535 and 80 participants with ${\dot{V}O}_{2}\max$ data at baseline (within the first week after diagnosis) were included from the intervention- and observational study, respectively. Three tests were excluded due to obvious technical issues (e.g. leakages from the face mask or technical errors), but otherwise, all available baseline ${\dot{V}O}_{2}\max$ tests were included in the analyses.

The Phys-Can intervention study was approved by the Regional Ethical Review Board in Uppsala, Sweden (Dnr 2014/249) and registered in ClinicalTrials.gov (TRN = NCT02473003, October, 2014).

### Cardiorespiratory fitness test

The participants were told not to eat, and drink anything other than water 2 hours before the test. In addition, they were told not to perform strenuous physical activity on the test day or the day before. At the test location, height and body mass were measured to the nearest 0.5 cm and 0.1 kg, respectively, while wearing light clothes and no shoes [43].

Participants performed a continuously graded exercise test on a motorized treadmill (In Uppsala; SportsArt Fitness Tr32, Washington, USA, in Lund; Rodby RL2500E, Vänge, Sweden and in Linkøping; GE T2100, Helsinki, Finland (in 2015) and Rodby RL2000, Vänge, Sweden (the remaining study period)) using a modified Balke protocol. Following a 5-min warm-up with increasing workload, participants started at 4 km/h with an inclination of 2%. The inclination increased with 2% each minute until reaching 12%, from which only the speed increased 0.5 km/h per minute until exhaustion [43]. Gas exchange data were obtained breath-by-breath, using the following different gas-analyzers: Uppsala; Viasys Vmax Encore, Care Fusion, San Diego, USA (accepted measurement errors for O_2_ analyzer: ±0.06–1%), Lund; Jaeger Oxycon Pro, CareFusion, Hoechberg, Germany (accepted measurement errors for O_2_ analyzer: ±0.05%) and in Linköping; Jaeger Oxycon Pro, CareFusion, Germany, Hoechberg (until Dec 15) and Cosmed Quark CPET, Rome, Italy (accepted measurement errors for O_2_ analyzer: ±0.02%) in the remaining study period. The software used was: Uppsala; Vmax Encore and Cardiosoft ECG, Version 6.7, San Diago, USA, Lund; LabManager, Jlab, CareFusion, version 5.31.0, Hoechberg, Germany and in Linköping; LabManager, Jlab, CareFusion, version 5.31.0.83, Hoechberg, Germany (in 2015) and Cosmed Quark PFT Ergo, Rome, Italy for the remaining study period. To assess the rate of perceived exertion (RPE), Borg’s scale_6-20_ was applied during and at the end of the ${\dot{V}O}_{2}\max$ test [44]. Instructions in how to use this scale were given before the test.

During the test, HR was measured using a Polar RS400 HR monitor in Uppsala, a Coded Polar receiver 4208 (connected to Oxycon Pro) in Lund and a heart rate receiver in the EKG equipment (GE Healthcare, CASE GE (connected to the Oxycon Pro) and a Cosmed SZ990 receiver (connected to the Cosmed Quark CPET) in Linköping. The peak average over 5 or 15 seconds was used when presenting HRpeak. Regarding ${\dot{V}O}_{2}$, RER and *f*_*R*_, the highest 60 s mean of the 10-, 15- or 30 s sampling averages (acquisition time differed between the tests/labs) in the last part of the test was reported as the peak value. When describing fulfillment of different percentages of predicted HR, the Tanaka equation, 208 − (0.7*age) was applied because this has been found to be more valid than the often-used 220 − age HRmax equation [45].

### Detecting a plateau in oxygen uptake

A computer program was developed to detect whether a ${\dot{V}O}_{2}$ plateau or leveling off occurred during the test time. Using this program, each of the extracted excel files with the test results were processed using an algorithm based on the definition of ${\dot{V}O}_{2}$ plateau by Taylor and colleagues [46], where a change in ${\dot{V}O}_{2}$ should be less than 150 mL from one minute to the next ($\Delta {\dot{V}O}_{2}$ ≤150 ml/min). Additionally, the cut-points of ≤80 ml/min and ≤50 ml/min were studied with similar definitions using the program. The highest average in ${\dot{V}O}_{2}$ over 1 minute was compared with the minute before or the minute after and whether ${\dot{V}O}_{2}$ for these time points differed ≤150 mL, ≤80 mL and ≤50 mL. Each of these three cut-points was investigated to descriptively present the prevalence of fulfilling each cut point. In the logistic regression analysis, the cut-point of ≤150 ml/min was chosen to be included because this is believed to fit best with our test-protocol which has very small expected ${\dot{V}O}_{2}$ increments between each stage [46].

### Test leader evaluation

After completing the tests, the test leaders were instructed to report factors related to challenges that could affect test outcomes. Additionally, each test leader reported the evaluation of every test with respect to whether the test was defined as “to exhaustion”. The evaluation was based on the observed body language, such as unsteady walking/running, bending the upper body (e.g. bending forward), facial expression showing exhaustion, hyperventilation and other signs reflecting that a maximal effort had been given. All test leaders were instructed, certified and followed up by the same person in the Phys-Can project group. A pilot-study was additionally conducted before the Phys-Can intervention study, where the predefined standards and test protocols were proven by the test leaders (and with some cancer patients).

### Participant characteristics and questionnaires

Living situation, education, sick-leave, smoking status and diagnosis were retrieved through questionnaires and medical journals. The Multidimensional Fatigue Inventory (MFI) [47] and European Organization for Research and Treatment of Cancer Quality of Life Questionnaire for Cancer patients (EORTC QLQ C30) [48] were used to retrieve information about physical fatigue, global health status and physical function.

### Physical activity monitoring

The number of hours in moderate to vigorous intensity physical activity per day was retrieved from the physical activity monitor SenseWear Armband Mini (BodyMedia Inc., Pittsburgh, PA, USA). The activity monitor was delivered on the day the ${\dot{V}O}_{2}\max$ test was performed. Patients were instructed to wear it for 7 consecutive days, accepting at least 4 days of registration with at least 80% wearing time each day. Physical activity registrations above 3 metabolic equivalents (METs) were defined as moderate to vigorous intensity physical activity [49].

### Statistical analyses

Patient characteristics and results from the ${\dot{V}O}_{2}\mathrm{peak}$ tests were presented as mean values ± standard deviation (SD) and numbers with percentages. For descriptive purposes, the mean ${\dot{V}O}_{2}\mathrm{peak}$ within “fulfillment” and “not fulfillment” of a variety of criteria and cut-points used in the literature were presented in a figure using GraphPad Prism version 7.00 for Windows (GraphPad Software, La Jolla California, USA, www.graphpad.com).

To determine associations between the criteria variables and the test leader’s evaluation, logistic regression analysis was performed using the Hosmer step-down procedure [50]. The key dependent variables included in the initial bivariate analysis were achievement of a ${\dot{V}O}_{2}$ plateau, HRpeak, RERpeak, Borgs’ RPEpeak and *f*_R_peak. In addition, ${\dot{V}O}_{2}\mathrm{peak}$, diagnosis, age, body mass and test time were included as adjusting variables. All variables significant at the 0.25 level were included in the final multivariate model. The odds ratios (ORs) and 95% confidence intervals (95%CIs) were calculated for 0.10 units regarding RERpeak. To investigate collinearity and interaction, pairwise correlations were performed for all the five key dependent variables in addition to ${\dot{V}O}_{2}\mathrm{peak}$ and test time. Furthermore, a receiver operating characteristic (ROC) analysis was performed to establish cut-points for variables associated with the test leader’s evaluation. These cut-points represented the point where the sensitivity and specificity were highest in correctly categorizing the test leader’s evaluation (“to exhaustion” or not). Finally, a cross-validation of the cut-points found in the ROC analysis was performed on the participants in the Phys-Can Cohort study, using a cross-table.

The analyses were performed using SPSS (IBM Corp. Released 2017. IBM SPSS Statistics for Windows, Version 25.0, IBM Corp., Armonk, NY, USA) and Statistical Analysis System (SAS version 9.1.3, SAS, North Carolina, USA). The level of statistical significance was set to 0.05.

## Results

Baseline characteristics of the participants in the intervention and in the cohort study are presented in Table 1. The two samples were comparable in respect to all characteristics, where mean age was 59 years and both samples included approximately 80% women with breast cancer, 15% men with prostate cancer and 4%–5% patients with colorectal cancer.

**Table 1:** Baseline characteristics of the participants in the Phys-Can Intervention study and the Phys-Can Cohort study, presented in mean (SD) or numbers (%).

	Phys-Can Intervention	Phys-Can Cohort
Number of subjects, n	535	80
Age, years, mean (SD)	59 (12)	59 (11)
Female, n (%)	430 (80)	67 (84)
Living with a partner, n (%)	402 (75)	58 (73)
Completed University, n (%)	309 (58)	39 (49)
Sick-leave, n (%)	180 (34)	22 (28)
100% sick leave, n (%)	150 (28)	19 (24)
Obesity (BMI≥30), n (%)	84 (16)	13 (16)
Current smoker, n (%)	19 (4)	4 (4)
Diagnosis, n (%)		
Breast cancer	421 (79)	66 (83)
Prostate cancer	93 (17)	10 (13)
Colorectal cancer	21 (4)	4 (5)
Physical fatigue, MFI, mean (SD)	11.2 (4)	11.7 (4)
EORTC QLQ C30, mean (SD)		
Global health status/QoL	66.3 (20.2)	70.7 (17.7)
Physical Function	88.5 (13.5)	90.0 (11.5)
MVPA, hours/day, mean (SD)	1.23 (0.8)	1.12 (0.6)

*Abbreviations*: BMI = Body Mass Index; MVPA = Moderate-to-vigorous intensity physical activity; QoL = Quality of life.

Financial situation: worst = 1, best = 10. MFI, Physical fatigue: 4 = low fatigue, 20 = high fatigue. Global health status/QoL: 0 = low quality of life, 100 = high quality of life. Physical Function: 0 = low/unhealthy level of functioning, 100 = high/healthy level of functioning. Moderate-to-vigorous intensity = physical activity at or above 3 metabolic equivalents (METs).

Peak values and test duration from the cardiorespiratory fitness test are given in Table 2. The prevalence of fulfilment of the three ${\dot{V}O}_{2}$ plateau criteria cut-points in the intervention and cohort study were: $\Delta {\dot{V}O}_{2}$ ≤150 ml/min; 90% and 86%, $\Delta {\dot{V}O}_{2}$ ≤80 ml/min; 63% and 65%, and $\Delta {\dot{V}O}_{2}$ ≤50 ml/min; 45% and 53%.

**Table 2:** Peak values and test-duration from the ${\dot{V}O}_{2}\max$-tests performed at baseline in the Phys-Can Intervention study and the Phys-Can Cohort study, presented in mean (SD).

	*Phys-Can Intervention*	*Phys-Can Cohort*
${\dot{V}O}_{2\mathrm{peak}}$, *ml/kg/min*	29.8 (7.3)	29.2 (7.1)
HR_peak_, beats/min	166 (19)	168 (19)
*Predicted HR*_*max*_, %*	99 (9.2)	100 (9.6)
*RER*_*peak*_, ${\dot{\mathrm{VC}}O}_{2}/{\dot{V}O}_{2}$	1.16 (0.10)	1.19 (0.11)
*VE*_*peak*_, *l/min*	79 (20)	79 (19)
f_*R peak*_, *breaths/min*	40 (7.8)	41 (6.4)
*Borg scale*, *RPE*_*6–20*_	17.9 (1.6)	17.0 (1.3)
*Test-duration*, *min*	9.9 (2.8)	9.4 (2.6)

*Abbreviations*: *f*_R_ = respiratory frequency; RER = Respiratory Exchange Ratio; ${\dot{\mathrm{VC}}O}_{2}$ = carbon dioxide production; ${\dot{V}O}_{2}$ = oxygen uptake; VE = ventilation. *Percentage of predicted maximal heart rate, by the Tanaka formula.

In the intervention study there were 465 (87%) and 70 (13%) tests evaluated as “to exhaustion” and “not to exhaustion”, respectively. The corresponding numbers were 76 (95%) and 4 (5%) in the cohort study. For the intervention study, ${\dot{V}O}_{2}peak$ was significantly (p<0.001) higher in the tests evaluated as “to exhaustion” (30.3 ml/kg/min, CI: 29.6–30.9) than “not to exhaustion” (26.6 ml/kg/min, CI: 24.9–28.3).

**Fig 1:**
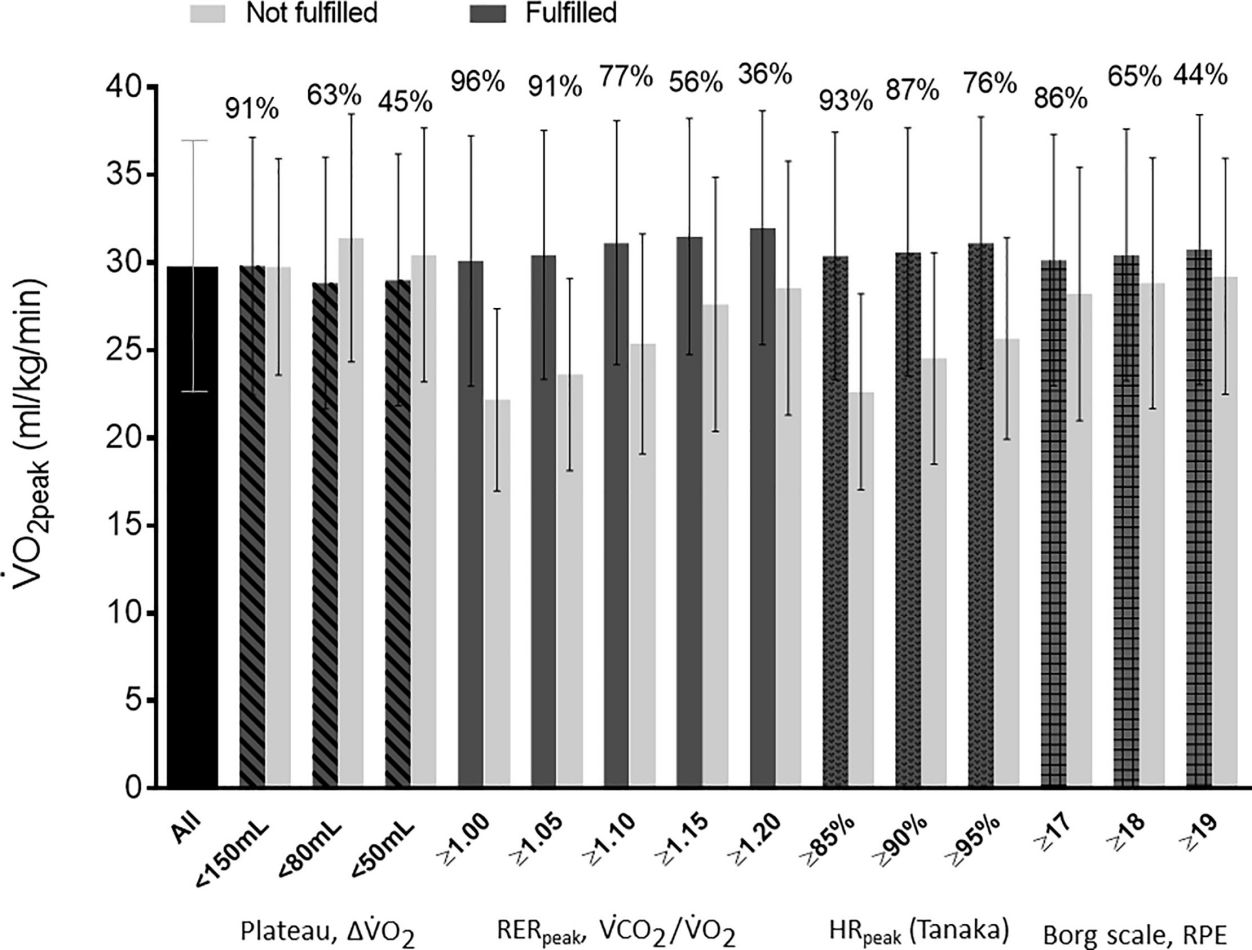
Mean (with SD) ${\dot{V}O}_{2}\mathrm{peak}$ stratified on fulfilling and not fulfilling criteria for ${\dot{V}O}_{2}\max$ max in patients diagnosed with breast, prostate or colorectal cancer (n = 535). *Abbreviations*: RER = Respiratory Exchange Ratio; RPE = rates of perceived exertion on Borg scale _6–20_; ${\dot{V}O}_{2}$ = oxygen uptake; ${\dot{V}\mathrm{CO}}_{2}$ = carbon dioxide production. Tanaka, HR_max_ = 208 - (0.7*age), Plateau, $\Delta {\dot{V}O}_{2}$ = a change in ${\dot{V}O}_{2}$ of less than 150, 80 or 50 ml/min from one minute to the next minute.

The percentage distribution and mean ${\dot{V}O}_{2}\mathrm{peak}$ in subjects fulfilling and not fulfilling different cut-points within the criteria of ${\dot{V}O}_{2}$ plateau, RER, predicted HR (Tanaka) and Borgs’ RPE are presented in Fig 1. Regarding the ${\dot{V}O}_{2}$ plateau criterion, the most accessible cut-point ($\Delta {\dot{V}O}_{2}$ ≤150 ml/min) was fulfilled by nearly all patients (91%), but mean ${\dot{V}O}_{2}\mathrm{peak}$ was the same as in patients who had not fulfilled this cut-point. The prevalence of fulfillment of cut-points was reduced by being stricter (≤80 [63%] and ≤50 ml/min [45%]), but mean ${\dot{V}O}_{2}\mathrm{peak}$ was significantly higher (p<0.001 and p = 0.028, respectively) in the patients who did not fulfill these two cut-points (Fig 1). The largest difference in ${\dot{V}O}_{2}\mathrm{peak}$ was observed between individuals who fulfilled (n = 514; 30.1 ml/kg/min) and those who did not fulfill (n = 21; 22.2 ml/kg/min) the RER≥1.0 criterion (p<0.001). Many patients fulfilled the strictest cut-point of ≥95% predicted HRpeak (76%). Regarding scoring on Borg’s scale, mean ${\dot{V}O}_{2}\mathrm{peak}$ in “fulfilled” vs “not fulfilled” did not differ across the three cut-points.

As seen in the bivariate analysis presented in Table 3, *f*_R_ peak, HRpeak, RERpeak, peak Borg’s RPE and plateau were significantly associated with the test leader’s evaluation (adjusted for age, diagnosis, ${\dot{V}O}_{2}\mathrm{peak}$ and test duration). Of the four adjusting variables, test duration was the only variable that was significantly associated to the test leader’s evaluation (p = 0.010). In the multivariate analysis, peak values for *f*_R_, RER and Borg’s RPE remained significantly associated with the test leader’s evaluation (Table 3). When adjusting for age, diagnosis, ${\dot{V}O}_{2}\mathrm{peak}$ and test duration, the probability of being categorized as “to exhaustion” was doubled both for each 0.1 increase in RER (OR: 2.07, 95%CI 1.39–3.08) and for each unit increase in Borg’s RPE (OR: 2.05, 95%CI 1.67–2.51). For each 10 breaths/min increase in *f*_R_, the probability of being categorized as “to exhaustion” was increased by 60%.

**Table 3:** Odds ratios (OR’s) from bivariate- and multivariate analysis with test-leaders’ subjective evaluation of the ${\dot{V}O}_{2}\max$ test as the outcome variable.

	*Bivariate analysis*		*Multivariate analysis*	
Effect variable	OR’s (95% CI)	p value	OR’s (95% CI)	p value
f_*R peak*_, *breaths/min*	1.12 (1.07, 1.17)	<0.001	1.06 (1.01, 1.12)	0.018
*HR*_*peak*_, *beat/min*	1.02 (1.00, 1.04)	0.017		
*RER*_*peak*_, ${\dot{\mathrm{VC}}O}_{2}/{\dot{V}O}_{2}$	2.21 (1.59, 3.08)	<0.001	2.07 (1.39, 3.08)	<0.001
*Borg scale*, *RPE*_*6–20*_	2.04 (1.68, 2.46)	<0.001	2.05 (1.67, 2.51)	<0.001
*Plateau, $\Delta {\dot{V}O}_{2}$ ≤150 ml/min*	2.22 (1.01, 4.87)	0.048		

The coefficients are given with 95% confidence intervals. *Abbreviations*: CI = Confidence interval; OR’s = Odds Ratio’s; *f*_R_ = respiratory frequency; HR = heart rate; RER = Respiratory Exchange Ratio; RPE = rates of perceived exertion; ${\dot{\mathrm{VC}}O}_{2}$ = carbon dioxide production; ${\dot{V}O}_{2}$ = oxygen uptake.

Adjusted for age, diagnosis, ${\dot{V}O}_{2}\mathrm{peak}$ and test-duration.

**Fig 2:**
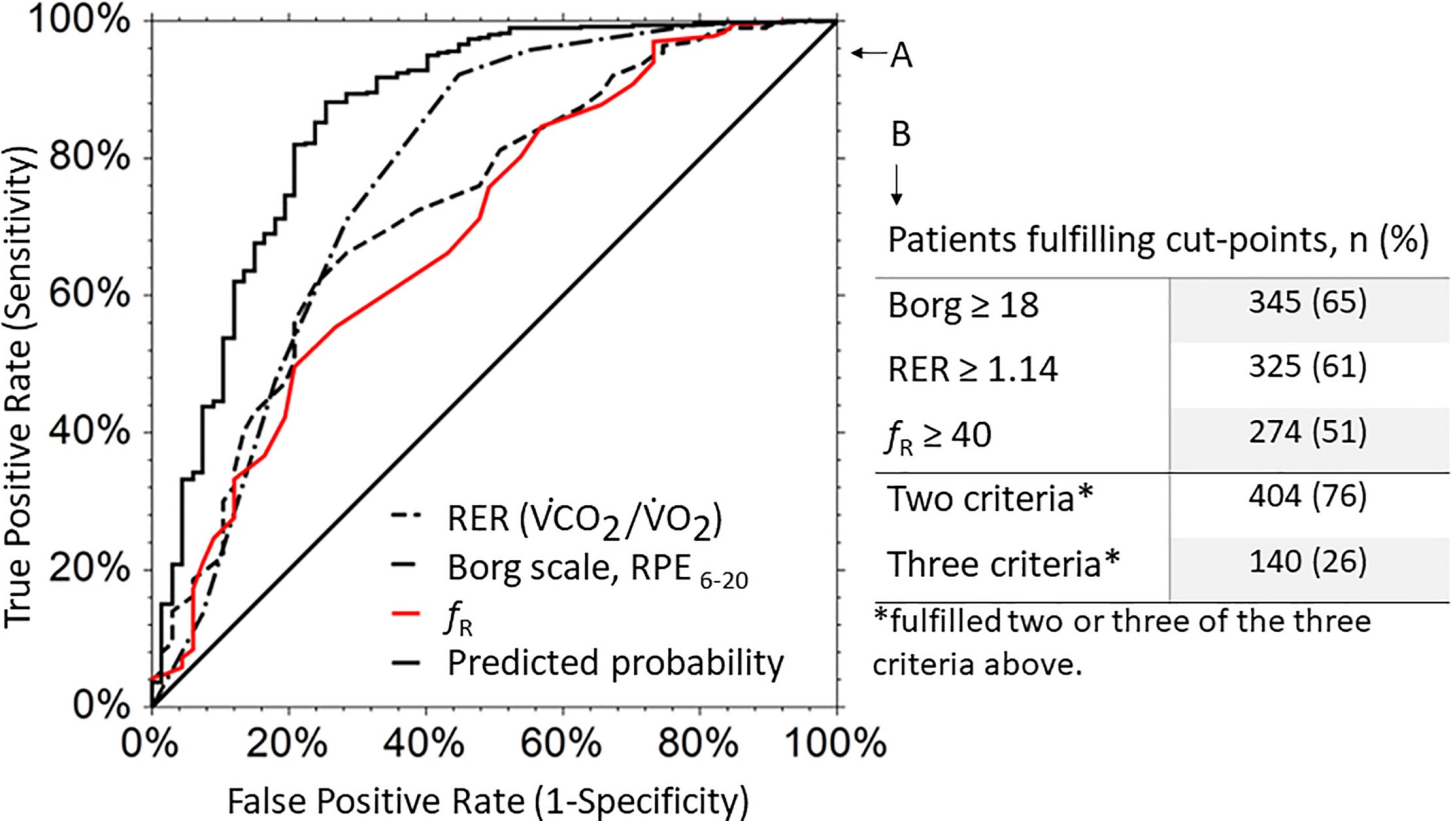
A: Receiver operating characteristic curves for RER, Borg’s RPE and *f*_R_, with the test-leader’s evaluation as the outcome variable. B: Number and percentage of patients fulfilling one, two and three of the criteria cut-points in the Phys-Can intervention study (n = 535). *Abbreviations*: RER = Respiratory Exchange Ratio; ROC = receiver operating characteristic; RPE = ratings of perceived exertion; *f*_R_ = respiratory frequency.

The three cut-points for these associated criteria, calculated from the ROC curves (Fig 2), were *f*_R_≥40 (true positive rate (TPR): 0.55, 95%CI 0.51–0.60), RER ≥ 1.14 (TPR: 0.66, 95% CI 0.62–0.70) and Borg≥18 (TPR: 0.71, 95% CI 0.67–0.75). The probabilities of correctly classifying the test leader’s evaluations were 77% for Borg’s RPE, 73% for RER and 70% for *f*_R_. When combining the three criteria, the predicted probability was the best (86%).

When performing the cross-validation analysis in the cohort study, three of the four (75%) tests classified as “not to exhaustion” were correctly classified. Regarding the tests classified as “to exhaustion” by the test leaders, 50 of the 76 tests (66%) were correctly classified. In total, 66% of the tests were correctly classified, and 34% were misclassified.

## Discussion

The criteria RERpeak, Borg’s RPE and *f*_R_ peak were associated with the test leader’s evaluation of whether a test was defined as “to exhaustion”. The cut-points that could best predict the test leader’s evaluation were RER≥1.14, RPE≥18 and *f*_R_≥40. Neither the HRmax criterion, nor attaining a ${\dot{V}O}_{2}$ plateau at the end of the ${\dot{V}O}_{2}\max$ test was associated with the test leader’s evaluation.

Of note, we observed that newly diagnosed cancer patients (before beginning treatment) responded similarly to healthy age-matched individuals in peak values of ${\dot{V}O}_{2}$, RER, Borg’s RPE and HR, although the present results are peak values (before applying any criteria verifying ${\dot{V}O}_{2}\max$) and the results from Edvardsen et al. were max values [51]. In addition, the cut-points of RER and RPE found through our ROC analysis did not differ from previously used cut-points in various populations [32, 52]. Therefore, we may assume that the cancer disease, per se, have not affected their ability to push themselves close to their maximal effort. Hence, the findings in the present study may be useful and transferable to other age-matched healthy individuals.

There is no “blueprint” regarding which outcome variable to apply when investigating criteria to verify ${\dot{V}O}_{2}\max$. Our experimental approach, in which the test leader’s evaluation is used for this purpose, has not been tried in this setting previously to our knowledge and is important to have in mind when interpreting our findings. Importantly, strong efforts were made in reducing the variation between test leaders through making the standards and protocols uniform for performing the tests, and all test leaders were certified by the same person who coordinated and ensured the quality of this part of the Phys-Can project.

### Respiratory exchange ratio

The RER≥1.14 cut-point that was determined through the ROC analysis, is similar to ≥1.15, which is a strict cut-point used in some studies [32], and to our knowledge, originates from the work by Issekutz et al from the 1960s [53]. In the present study, a finding of 56% participants fulfilling the ≥1.15 criterion, was in agreement with Edvardsen and colleagues’ participants (aged 20–85 years), where 65% achieved this cut-point [37], especially when taking age into consideration. In a study of younger (mean age 37 years) overweight and obese adults, the prevalence of achieving RER≥1.15 was higher (89%) [38]. In similar treadmill protocols, RERpeak was found to decrease with age [37], and considering that our participants had a mean age of 59 years, the mean RERpeak of 1.16 in the present study was comparable to the mean RERpeak of 1.17 seen in participants from 50 to 64 years old in Edvardsen and colleagues’ study [37]. Nearly all subjects (96%) in the present study fulfilled the RER≥1.0 criterion and 91% reached the age-related recommended cut-point of RER≥1.05 for healthy individuals [37]. Schneider et al. (2019) [42] found percentage of fulfillment of the RER≥1.1 cut-point (84%) to be similar as in the present study (77%), though slightly higher, possibly because of using a cycle ergometer.

In healthy and clinical populations, the rationale for choosing one cut-point instead of another seems to be lacking, and because several cut-points have been used previously, ranging from 1.00 to 1.20 [52], the selected cut-points may have been arbitrary [35]. Explanations for why people attain different levels of RERpeak at maximal tests are not fully understood, but age may affect RERmax [37]. Another factor is the test protocol used. Because a more rapid incremental work rate increases the anaerobic energy contribution, the rate of HCO_3_ buffering of lactic acid-derived H^+^ ions is increased (i.e. the rate of CO_2_ output will be greater because it follows the rate of H^+^ buffering) [54]. Consequently, shorter and faster test protocols result in higher RERpeak values compared with ramp tests that are of longer durations [35]. The RER cut-off values should therefore probably be made protocol specific.

Food intake and medication are also important factors that may affect RERpeak. It was suggested that habitual dietary patterns that influence the systemic acid load may account for 19% of the variability observed in RERpeak [55]. In women treated with chemotherapy and tamoxifen-like drugs, the accumulation of lactate was less compared with healthy women, especially at high exercise intensity (70% of ${\dot{V}O}_{2}\max$) [56]. In combination with the observed lower carbohydrate oxidation and greater fat oxidation, the authors suggested that the cancer itself, and/or the medications received, may disrupt normal energy metabolism in patients with cancer during exercise [56]. This highlights the importance of validating these criteria in different patient groups, and in cancer patients the validation should also be made in tests completed during treatment.

### Perceived exertion

A Borg’s RPE of ≥18, found in our ROC analysis, did not differ from cut-points often seen in the literature, with observed cut-points of ≥17, ≥18 or ≥19 [52]. Congruent with our observations, 84% of participants in Edvardsen et al. (2014) achieved the most frequently used cut-point of RPE≥17. Despite close relationships between scores on Borg’s scale and physiological measures of intensity, such as HR, BLa^-^ [57], and work rate during exercise [58], the validity of Borg’s scale as a criterion in ${\dot{V}O}_{2}\max$ testing has been questioned [59]. The validity in the use of this criterion depends on the subject’s understanding of the scale and associated verbal descriptors, the ability to differentiate between discomfort and physiological fatigue and motivation [60]. It has been proposed that physically inactive individuals not accustomed to exercise until exhaustion are likely to report perceived maximal exertion before they actually reach their true ${\dot{V}O}_{2}\max$ [21]. The discrepancy between the percent of participants reaching RPE≥17 (86%; 30.2 ml/kg/min) and ≥18 (65%; 30.4 ml/kg/min) was large in our study, congruent with no differences in ${\dot{V}O}_{2}\mathrm{peak}$ within fulfilling the two cut-points. Consequently, choosing an RPE≥17 cut-point would probably also work well for this patient group.

### Respiratory frequency

Through the ROC analysis, ≥40 breaths/min was found to be the cut-point best associated with the test leader’s evaluation. This cut-point was reached by 52% of the participants, and these participants had a significantly (p<0.001) higher ${\dot{V}O}_{2}\mathrm{peak}$ (32 ml/kg/min), than participants not achieving this cut-point (27 ml/kg/min). To our knowledge, *f*_R_ has not been used as a criterion verifying ${\dot{V}O}_{2}\max$ in previous studies, but there are implications that *f*_R_ is a potentially valid measure that reflects physical effort. In two studies by Nicolo et al. [40, 61], the authors describe why *f*_R_ is a better marker of physiological strain compared with the variables ${\dot{V}O}_{2}$, HR and BLa^-^. The nonlinear increase of *f*_R_ during incremental exercise follows the level of acidosis from lactate production and is not affected by muscle damage or glycogen depletion, suggesting that physical effort is more causally linked with *f*_R_ than BLa^-^. In addition, *f*_R_ is closely related to RPE in fit males (20±3 years) and does not seem to be affected by choice of test protocol [61]. Whether *f*_R_ is a valid criterion to apply as part of verifying ${\dot{V}O}_{2}\max$ needs to be investigated in future studies.

### Age predicted maximal heart rate

The age predicted HRmax was not significantly associated to the test leader’s evaluation of whether the test was performed “to exhaustion”. In ${\dot{V}O}_{2}\max$ tests performed in different populations, fulfillment of various cut-points representing percentages of age predicted HRmax are often seen [39, 62]. Because of 10- to 12-beats-per-minute variations in HRmax in healthy individuals, even when taking age into account [63, 64], predicting HRmax is problematic [65, 66], and is likely to underestimate or overestimate HRmax on an individual level. A potentially greater variation is added in patients with cancer owing to the documented impact certain cancer treatments have on cardiac function [67], which is commonly observed as increased HR [68]. In addition, on the basis of the possible positive effects of beta-blockers (which cause lower HR or a “ceiling” in HR) in relation to cancer prognosis [69], such medications also contribute to complicating the use of this criterion. Taking these factors together, the age predicted HRmax is presumably a problematic criterion to apply in both healthy individuals [22, 39] and in patients with cancer, before, during and after cancer treatment.

### Plateau in oxygen uptake

Finding as many as 91% to achieve the ≤150 ml/min plateau cut-point may be interpreted as a positive finding. However, the mean ${\dot{V}O}_{2}\mathrm{peak}$ was the same as in the patients that did not fulfill this cut-point. Whether or not ≤150 ml/min plateau cut-point fits the participants and protocol in the present study, could be discussed. The modified Balke protocol involves very small ${\dot{V}O}_{2}$-increments from one stage to the next, and therefor seems the most suitable for the 150 ml/min cut-point, compared to the other two cut-points applied in the present study. A plateau in ${\dot{V}O}_{2}$ stands out as the most widely used criterion for verifying ${\dot{V}O}_{2}\max$ [23], but some authors doubt that such a physiological plateau exists [30]. Others argue that a ${\dot{V}O}_{2}$ plateau exists, but the methodology used to identify it is central for detecting it [36]. The type of test protocol and sampling acquisition may affect the observation of a plateau [31, 32], in addition to age and fitness [23], although other studies do not agree on this [33]. Although researchers do not agree on the rationale, undoubtedly there are huge variations in the number of subjects fulfilling the plateau criterion in different studies [32]. Based on all considerations, questions are raised regarding the validity of using the plateau criterion verifying true $\dot{V}O_{2}\max$ [70] and other researchers have concluded that the $\dot{V}O_{2}$ plateau is not a reliable physiological marker for maximal effort in all subjects [71].

### Strengths

In a large sample of patients newly diagnosed with cancer, we have managed to elucidate criteria for validating ${\dot{V}O}_{2}\max$ tests differently from what has been previously seen in the literature. Thorough and consistent instructions and follow-up of the test leaders enabled conditions to be as similar as practically possible for all participants, independent of when or where they performed their ${\dot{V}O}_{2}\max$ tests. The test leaders were also generally experienced with exercise testing and/or with the clinical populations before the start of the Phys-Can. By including *f*_R_ in our analyses, we have started to explore another possible variable as a new criterion or normative to apply in validation of ${\dot{V}O}_{2}\max$ tests.

### Limitations

Few patients with colorectal cancer were included, so generalization to this or other nonincluded types of cancer are questionable. Furthermore, because there were only 4 of 80 (5%) ${\dot{V}O}_{2}\max$ tests evaluated as “not to exhaustion” in the cohort study, our cross-validation was more of a descriptive approach. The O_2_ analyzers were from different producers across the three sites, and this may be a source of bias between the tests performed in Lund, Linköping and Uppsala. For practical reasons, validity tests were, unfortunately, not performed between the various O_2_ analyzers. Measurements of BLa^-^ were not taken after the ${\dot{V}O}_{2}\max$ tests in the Phys-Can study. Although the RER value correlates highly with BLa^-^ [72], a measure of BLa^-^ would have expanded the number of objective criteria assessed. In addition, high inter-subject variability (from 5 to 17 mM) in post-exercise lactate has been reported [73] and is, accordingly, another criterion that is difficult to standardize [35]. The definition of a ${\dot{V}O}_{2}$ plateau, as included in the present study, is perhaps not the most suitable method because of the protocol-differences between the discontinuous test protocols applied on healthy young men in the 1950s by Taylor et al. and the modified Balke protocol used in Phys-Can. In addition, we did not incorporate relative body mass into the equation. The validity of the results from the cross-validation, where a correct classification of “to exhaustion” were made in only 66% of cases from the cohort study, when applying the best three criteria can be questioned. However, the low number of tests classified as “not to exhaustion” in the cohort study makes the data figures too small to conclude anything related to how well the criteria fits another comparable sample of individuals. Last, in the present study we did not include a verification bout directly after each of the ${\dot{V}O}_{2}\max$ tests, which potentially could have been a better approach than the test-leaders evaluation as the effect variable when investigating the different criteria and their cut-points.

### Conclusions and future perspectives

Relating the findings to clinical practice, we suggest avoiding the predicted HRmax criterion. On the basis of the observations in the present study, in addition to the complexity of detecting a ${\dot{V}O}_{2}$ plateau when using different methodologies (e.g. test protocols and data acquisition) [23], we suggest not placing emphasis on this criterion either. We recommend a focus on RER (in the range between ≥1.1 and ≥1.15) and RPE (≥17 or ≥18) in addition to the test leader’s evaluation. Also, a *f*_R_ peak of ≥40 breaths/min may be an additional cut-point to help the test leader evaluate the degree of exhaustion, but more research is needed to determine whether this should be used as a criterion.

A course for future investigations may be to determine whether the *f*_R_ variable could be part of the criteria verifying ${\dot{V}O}_{2}\max$. In addition, it would be interesting to precede with comparable methodologic approaches as in Schneider et al. (2019) [42], where a supramaximal verification bout was performed after the ${\dot{V}O}_{2}\max$ test, in order to validate the initial ${\dot{V}O}_{2}\max$ results, only apply the method using treadmill [20]. Also, a submaximal verification phase [36] which probably is more feasible for cancer patients, would be interesting to apply and investigate further. Whether achievement of the same ${\dot{V}O}_{2}\max$ value in the verification bout is a valid criterion could be investigated together with the results from the present study, in patients in different phases of their cancer disease. In a recent study by Santa Mina et al. (2020), the authors describe their lab-experiences from testing 44 patients with cancer, in which only 14% achieved all of their ${\dot{V}O}_{2}\max$ criteria, and none reached a ${\dot{V}O}_{2}$ plateau [74]. Hence, it is also important to investigate criteria for verifying ${\dot{V}O}_{2}\max$ in patients that are undergoing or have finished cancer treatment, as these patients may have other responses and may have more difficulties in pushing themselves to maximal effort.

## Supporting information

S1 Dataset (XLSX)
